# Physicians Treating Alzheimer’s Disease Patients Should Be Aware that Televised Direct-to-Consumer Advertising Links More Strongly to Drug Utilization in Older Patients

**DOI:** 10.3233/JAD-210294

**Published:** 2021-06-01

**Authors:** Robin Feldman

**Affiliations:** Arthur J. Goldberg Distinguished Professor of Law and Director of the Center for Innovation at University of California, Hastings Law, San Francisco, CA, USA

**Keywords:** Aged, direct-to-consumer advertising, drug utilization, health services for the aged, prescription drugs, public health, television

## Abstract

**Background:**

US direct-to-consumer advertising spending for medicine has soared in recent decades. Advertising has been shown to impact drug utilization. Most Alzheimer’s disease patients are above age 65 and may take a range of prescription medications for various disease states.

**Objective:**

To investigate how direct-to-consumer advertising is associated with the drug utilization of patients ≥65 years old.

**Methods:**

Using advertising expenditure data and Medicare Part D drug purchase claims, we performed regression analyses for each of the highest-spending drugs and age group, with cumulative monthly spending as the predictor variable and drug utilization as the response variable. For each drug, we ran a second set of regression analyses to determine if the spending-utilization correlation showed a significant difference between the two patient age groups (older than 65, younger than 65).

**Results:**

For all 14 drugs in our study, advertising spending is positively correlated with utilization (*p* < 0.01) in both age groups. For seven of the 14 drugs studied, the difference in the utilization of patients older than 65 and the utilization of patients younger than 65 is statistically significant at a *p* < 0.01 level. The 65-and-older age bracket exhibits significantly greater utilization for all seven of these drugs.

**Conclusion:**

We find televised advertising for certain drugs to be associated with significantly stronger drug utilization among seniors, as compared to younger patients. Alzheimer’s disease physicians should be aware of this result, in light of the medications that patients may take for other disease states, particularly mood and mental health medications.

## INTRODUCTION

Direct-to-consumer advertising (DTCA) is the marketing of pharmaceutical products directly to patients, rather than health care professionals. The United States and New Zealand are the only countries that fully permit this practice, which spans print, radio, Internet, and television programming [1]. Direct-to-consumer advertising spending has soared in recent decades [2]. Between 1997 and 2016, the pharmaceutical industry increased its annual direct-to-consumer advertising spend from $2.1B to $9.6B, a surge that included an eightfold increase in the total number of television ads [2].

Debates about direct-to-consumer advertising are well-attended on both sides. Some proponents claim that direct-to-consumer advertising lowers prescription drug prices by increasing competition [1]. By offering information about available treatments, adv-ertising may empower patients to engage in educated discussions with health care professionals or improve health outcomes by encouraging patients to begin utilizing appropriate drugs more quickly [1, 3]. Dir-ect-to-consumer advertising has also been shown to increase physician visits [4] and improve patient compliance for certain medications [5].

On the other hand, advertising to patients rather than health care professionals may raise ethical and regulatory concerns. Critics of the practice charge that direct-to-consumer advertising contributes to prescription drug overuse and disease mongering [6], increasing health care spending in the process. For some conditions, direct-to-consumer advertising has increased inappropriate prescriptions [7], resulting in more adverse drug reactions [8].

Direct-to-consumer advertising patterns suggest that the activity is unlikely to play a positive role in promoting competition or lowering prices. In general, pharmaceutical companies tend to use direct-to-con-sumer advertising to promote drugs without competitors in the market [9]. When no competitor exists, advertising would do little to promote competition. Advertising for a drug without competition may inform patients about an innovative treatment option [1], but advertising in this context may also help create or maintain patient loyalty toward pharmaceutical products that are not strictly necessary for their ailment [6]. Moreover, advertising costs are simply passed on to the patient in the form of higher prices [10, 11].

When pharmaceutical companies do use direct-to-consumer advertising in markets characterized by both a brand and a generic version, the effects are also unlikely to enhance competition and lower pri-ces. Generic companies generally do not engage in direct-to-consumer advertising, given their low profit margins [12]. Thus, advertising for the brand alternative serves to push consumers toward the more expensive choice, limiting the competitive benefit of generics on the market [10]. Soaring advertisement spending may also divert financial resources away from pharmaceutical research and development [11].

Regulation of direct-to-consumer advertising in the United States is limited. Although most advertising is regulated by the competition agency, the Federal Trade Commission, advertising of prescription drugs is regulated by the U.S. Food and Drug Administration (FDA) [1, 11]. The FDA requires that drug advertisements convey a balanced disclosure of a medication’s benefits and risks [10]. Pharmaceutical companies assert, however, that freedom of speech insulates them from many content restrictions, and the FDA to date has avoided wading into regulating anything beyond such disclosures [13].

Tax rules also provide advantages for direct-to-consumer advertising over other types of promotional activities. In contrast to other pharmaceutical promotion practices that are subject to spending caps, such as physician detailing, direct-to-consumer advertisement spending may be claimed as a tax deduction [10, 14].

Most important, past research demonstrates that direct-to-consumer advertising increases patient drug utilization along two avenues. Some studies have shown that advertising spending initiates new doctor visits [4, 15], netting new prescriptions for cer-tain drug classes [16, 17]. Other work has found that advertising increases patient willingness to fill existing drug prescriptions [17]. Looking more broadly at spending and utilization, direct-to-consumer advertising has been shown simply to increase an adver-tised drug’s sales [18]. To this end, some analyses have attributed as much as 20–30%of American prescription drug expenditure growth to direct-to-consumer advertisement spending [17, 18]. Despite this considerable work regarding the effects of advertisement on drug utilization, however, the impact on the drug utilization behaviors of senior citizens constitutes a gap in the empirical literature. Our study aims to help fill this gap.

Specifically, our study focuses on television to examine how advertising shapes the drug utilization of seniors. Since the FDA relaxed advertising content regulations in 1997, television has become the principal medium of direct-to-consumer advertising [1]. Television is particularly popular among senior citizens (65 and older), who on average watch up to two more hours of television daily than younger Americans [19]. Finally, we focus on television because it is a highly effective advertising medium: One study of pharmaceutical spending found that broadcast advertising, which includes television ads, boosted drug sales with twice the elasticity of non-broadcast advertising [18].

Evidence of age-related declines in decision-making and information processing raises particular consumer protection concerns related to direct-to-consumer advertising. Past surveys of older adults exposed to direct-to-consumer advertising found that older adults tend to notice and retain less information about the advertised drug compared to younger patients, including details about side effects, risks, and whether the drug requires a prescription [20]. Older adults also tend to display poorer health numeracy [21] and health literacy [22]. Patients with lower health literacy, in turn, are less likely to recognize the names or purposes of medications they are prescribed [23].

Older adults also are more susceptible to “illusions of truth”, in which repeated false information is believed true [24]. One study found that older adults were more likely to misremember repeated product warning claims as recommendations [25], suggesting that advertising risk and benefits disclosures may offer limited consumer protection benefit to the elderly. Moreover, source, or context, memory declines with age [25], meaning that seniors may be at greater risk of recalling information dispensed by a paid spokesperson as advice from a trusted health care professional.

Qualities of television advertising may compound cognitive deficits in older adults, so that prescription drug marketing may be disproportionately effective among older populations. For instance, the growing frequency of televised drug ads [1, 2] could exacerbate seniors’ illusions of truth. The imagery of a positive, post-treatment patient experience that in-creasingly dominates televised prescription drug ads [26] may complement the tendency of seniors to pri-vilege positive information when engaged in deci-sion-making [27]. Related to this point, past research demonstrates that older adults, especially those with cognitive deficits, consistently judge medications more favorably when they are positively framed [28].

Finally, seniors are major stakeholders in the pharmaceutical advertising conversation. The likelihood of having a drug prescription increases with age [20], as does the number of annual prescriptions per individual [29]. Moreover, elderly prescription drug usage—measured both in terms of median prescriptions per individual and the proportion of older adu-lts taking 5 or more medications—has grown dramatically in recent decades [30]. The 2006 inauguration of the Medicare Part D program, which offered prescription drug coverage to all Americans over 65, has contributed to this trend [30], boosting elderly prescription drug utilization rates by 12.8%[31]. Pharmaceutical companies seem to understand elderly usage patterns well and have used television to capitalize on those patterns. For example, one analysis found that in the months following the passage of Medicare Part D, elderly-dense areas experienced greater exposure to televised pharmaceutical advertising compared to areas with a younger population [17].

With these advertising practices in mind, we att-empt to examine how direct-to-consumer advertising is associated with seniors’ drug utilization. Past research demonstrates that direct-to-consumer advertising spending increases utilization of advertised drugs [5, 15–18]. Our study does not attempt to show that televised advertising spending causes stronger drug utilization responses in seniors. Rather, we build on these past analyses of advertising spending to investigate associations between televised advertising spending and drug utilization by patients older than 65 [5, 15–18].

## METHODS

### Data source for televised direct-to-consumer advertising spending

We licensed advertisement expenditure and occurrence data from a media analytics agency, Kantar Media. We then performed regression analyses on trend reports generated from this data, filtering for network and cable television advertising of pharmaceutical products between 2006–2017. Trend reports returned the name of the advertised prescription drug, along with the name of its manufacturer, its treatment group, and the monthly amount spent on its televised advertisement.

### Data source for prescription drug utilization

We purchased data from the Centers for Medicare and Medicaid Services on a cohort of roughly one million Medicare patients in order to examine the cohort’s Part D drug purchase claims filed during the period of 2006–2017. To qualify for Medicare, one must be 65 years or older, or be younger than 65 years old, and suffer from: a permanent disability, amyotrophic lateral sclerosis, or permanent kidney failure [32]. In 2006, the passage of Part D expanded Medicare services to include prescription drug coverage [31].

All patients included in our cohort were enrolled only in Medicare Part A (hospital), B (doctor & outpatient), and D (prescription drug) coverage, excluding Part C. While Part C (Medicare Advantage) plans may also cover prescription drugs, the extent of coverage is not uniform between plans, and some Part C plans do not cover prescription drugs at all [33]. The Part D dataset only included patients who experienced at least one prescription drug event and remained alive between 2006–2017. A prescription drug event is a single instance of a patient filling a prescription for a drug with exactly one corresponding National Drug Code.

We divided our claims data into two patient subpopulations. Patients younger than 65 comprised 35.7%of the total patient population, while patients 65 and older accounted for the remaining 64.3%. We collapsed and aggregated the individual claims data to reflect monthly utilization of each drug for each age group. Monthly drug utilization refers to the number of patients who used a specific drug in one month.

### Prescription drug selection

We further filtered the trend reports generated from Kantar to identify the five treatment groups with the highest televised direct-to-consumer advertising spending between 2006 and 2017. Although the Impotence treatment group ranked second in total televised advertising spending, we did not analyze drugs in this group because Medicare Part D generally does not cover impotence medications [34]. To clarify our categories, we combined the drugs treating depression and bipolar disorder into one category—Mood and Mental Health. Consequently, our study drew from four treatment groups: Arthritis, Diabetes (Non-Insulin), High Cholesterol, and the combined Mood and Mental Health category. We selected the prescription drugs from these four groups whose cumulative ad spending during the study period exceeded $150 M (Table 1). We elected to focus on high-spending drugs because, were we to find that high-spending drugs are not associated with greater drug utilization, then it would be reasonable to conclude that lower-spending drugs likely bore little influence on drug utilization, either. We note that few drugs for the treatment of Alzheimer’s disease have direct-to-consumer advertising during the study period, and the advertising spending is relatively low for those that do.

**Table 1:** Drugs exceeding cumulative televised direct-to-consumer spending of $150M from treatment groups with highest televised direct-to-consumer spending (2006–2017)

	Trade Name	Generic Name	Drug Manufacturer	Treatment Group	Cumulative Spending
1	**Latuda**	Lurasidone	Sumitomo Dainippon Pharma	Mood and Mental Health	$321.5M
2	**Abilify**	Aripiprazole	Otsuka Pharmaceutical	Mood and Mental Health	$528M
3	**Cymbalta**	Duloxetine	Eli Lilly	Mood and Mental Health	$785M
4	**Humira**	Adalimumab	AbbVie	Arthritis	$1.5B
5	**Enbrel**	Etanercept	Amgen	Arthritis	$662.5M
6	**Xeljanz**	Tofacitinib	Pfizer	Arthritis	$471.6M
7	**Celebrex**	Celecoxib	Pfizer	Arthritis	$447.5M
8	**Trulicity**	Dulaglutide	Eli Lilly	Diabetes	$222.9M
9	**Victoza**	Liraglutide	Novo Nordisk	Diabetes	$267.4M
10	**Farxiga**	Dapagliflozin	AstraZeneca	Diabetes	$265.2M
11	**Jardiance**	Empagliflozin	Boehringer Ingelheim	Diabetes	$152.2M
12	**Invokana**	Canagliflozin	Johnson &Johnson	Diabetes	$192.9M
13	**Lipitor**	Atorvastatin	Pfizer	High Cholesterol	$665.5M
14	**Crestor**	Rosuvastatin	AstraZeneca	High Cholesterol	$472.4M
	Total				$7.0B

Our model only included the period of time when, for each drug, no generic competitor was present. Generic competition negatively and significantly impacts branded drug utilization, which could provide a confounding factor in the results [31]. Moreover, drug companies generally stop or reduce advertising spending as generic competition arrives on the market [9]. We defined a generic competitor as a drug produced by another manufacturer with the same active ingredient, dosage, and delivery method. In the High Cholesterol treatment group, rosuvastatin and atorvastatin are both high-intensity statins [32]. To provide consistency within the model, we stopped considering both rosuvastatin and atorvastatin when a generic atorvastatin entered the market in 2011 (Table 2).

**Table 2:** Summary of findings

Drug Name^*^	Period of Televised Advertising^†^	Significance (*p* < 0.01)	Utilization			Patient Group with Greater Percentage Change	Percentage Change *X*-Times
			General	Older	Younger
				than 65	than 65
Lurasidone^‡^	Jan. 2014 –Dec. 2017	***Yes***	Increasing	Increasing	Increasing	Older than 65	***3.5***
Aripiprazole	Sep. 2007 –Mar. 2015	***Yes***	Increasing	Increasing	Decreasing	Older than 65	***2.5***
Duloxetine	Apr. 2006 –Nov. 2013	***Yes***	Increasing	Increasing	Increasing	Older than 65	***1.6***
Adalimumab	Jan. 2007 –Dec. 2014	***Yes***	Increasing	Increasing	Increasing	Older than 65	***1.7***
Etanercept	Aug. 2006 –Dec. 2016	***Yes***	Decreasing	Decreasing	Decreasing	Younger than 65	***1.2***
Tofacitinib	June 2013 –Dec. 2017	No	Increasing	Increasing	Increasing	Similar	-
Celecoxib §	Apr. 2007 –Nov. 2014	***Yes***	Decreasing	Decreasing	Decreasing	Younger than 65	***3.3***
Dulaglutide	Oct. 2015 –Dec. 2017	No	Increasing	Increasing	Increasing	Similar	-
Liraglutide	Sep. 2013 –Dec. 2017	No	Increasing	Increasing	Increasing	Similar	-
Dapagliflozin	Sep. 2014 –Dec. 2017	No	Increasing	Increasing	Increasing	Similar	-
Empagliflozin	Sep. 2015 –Dec. 2017	No	Increasing	Increasing	Increasing	Similar	-
Canagliflozin	June 2014 –Nov. 2017	No	Increasing	Increasing	Increasing	Similar	-
Atorvastatin	Mar. 2006 –Sep. 2011	***Yes***	Decreasing	Decreasing	Decreasing	Younger than 65	***1.1***
Rosuvastatin	Mar. 2006 –Sep. 2011^**^	No	Increasing	Increasing	Increasing	Similar	-

^*^Drug names are color-coded by treatment group, with Mood & Mental Health (pink), Arthritis (green), Diabetes Non-Insulin (yellow), and High Cholesterol (grey). ^†^The time period that our regression models covered started from the first occurrence of televised ad spending for a given drug and ended when spending ceased or when generic competition for the drug entered the market. Spending on televised ads for the vast majority of brand drugs ended with the entrance of a competing generic. Generic competition also has a negative and significant impact on the brand utilization; therefore, regardless of the magnitude of ad expenditures, utilization for a brand drug drops when there is a competing generic. ^‡^How to read: lurasidone is a drug in the mood and mental health treatment group. The total number of patients on lurasidone increased during this period, and the response percentage of older patients on lurasidone was 3.5 times higher than the response percentage of younger patients on lurasidone. §How to read: celecoxib is a drug in the arthritis treatment group. The total number of patients on celecoxib decreased during this period, and the response percentage of younger patients on celecoxib was 3.3 times lower than the response percentage of older patients on celecoxib. ^**^We assumed that rosuvastatin and atorvastatin were replaceable, since both drugs are high-intensity statins. Because atorvastatin had a generic in the market much earlier than rosuvastatin, we considered atorvastatin’s generic drug to be a competitor to rosuvastatin as well and therefore, considered rosuvastatin’s “end date” to coincide with the entrance of atorvastatin’s generic.

**Fig. 1:**
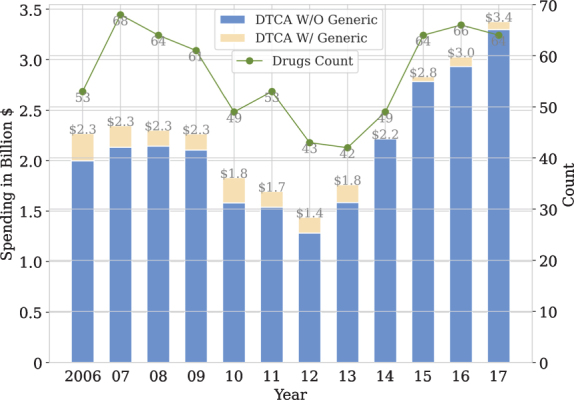
Direct-to-consumer televised advertising spending, 2006–2017. ^††^ Graph shows total direct-to-consumer televised ad spending for drugs with and without generic competitors, along with total number of advertised drugs during the study period. By including the drug count, we show that overall spending has increased due to greater spending per drug, rather than a greater number of advertised drugs.

In total, the 14 drugs we studied accounted for more than $7 billion in cumulative televised advertising spending between 2006–2017 (Table 1). This constitutes 25.6%of all televised pharmaceutical advertising spending during this period (Fig. 1).

### Statistical analysis

First, we sought to determine the strength and character of the correlation between cumulative televised advertising spending and the number of patients utilizing the advertised drug each month. For each of the 14 drugs and for both patient subpopulations, we performed regression analyses with cumulative monthly spending as the predictor variable and drug utilization as the response variable. Given that the response variable is a discrete variable, we opted to use Maximum Likelihood Estimation (MLE) rather than an Ordinary Least Squares (OLS) regression. Due to overdispersion in our initial Poisson regression models, we ran negative binomial regressions and explored both linear and nonlinear relationships.

We chose between the linear (Formula 1) and non-linear (Formula 2) models by performing a one-si-ded Chi Squared Cumulative Density Function test using the SciPy package in Python. If the inclusion of the quadratic term was statistically significant at a *p* < 0.01 level, we proceeded with the nonlinear model; otherwise, we proceeded with the linear model. Advertising spending values were divided by 1000 to make quadratic terms feasible for each regression model (Supplementary Table 1).

$$\begin{array}{cc} & \mathrm{Formula}\;1.\;\mathrm{In}\;(\mathrm{Patient}\;\mathrm{Count}\;\mathrm{for}\;a\;\mathrm{given}\;\mathrm{age}\\ & \;\mathrm{group})\;=\;\alpha \;+\;\beta \;\mathrm{DTCA}\;\mathrm{Spending}\;+\;ɛ \end{array}$$

$$\begin{array}{cc} & \mathrm{Formula}\;2.\;\mathrm{In}\;(\mathrm{Patient}\;\mathrm{Count}\;\mathrm{for}\;a\;\mathrm{given}\;\mathrm{age}\\ & \;\mathrm{group})\;=\;\alpha \;+\;\beta_{1}\;\mathrm{DTCA}\;\mathrm{Spending}\\ & \;+\;\beta_{2}\;\mathrm{DTCA}\;\mathrm{Spendin}g^{2}\;+\;ɛ \end{array}$$

*Where DTCA Spending is cumulative and monthly*

Next, we ran a second set of negative binomial regressions (Formula 3) for each drug to determine if the correlation between televised advertising spending and utilization was significantly different between the two patient subpopulations.

$$\begin{array}{cc} & \mathrm{Formula}\;3.\;\mathrm{In}\;(\mathrm{Patient}\;\mathrm{Count})\\ & \;=\;\alpha \;+\beta_{1}\;\mathrm{Dummy}\;+\;\beta_{2}\;\mathrm{DTCA}\;\mathrm{Spending}\\ & \;+\;\beta_{3}\;\mathrm{Dummy}\;*\;\mathrm{DTCA}\;\mathrm{Spending}\\ & \;+\;\beta_{4}\;\mathrm{DTCA}\;\mathrm{Spendin}g^{2}\;+\;ɛ \end{array}$$

*Where DTCA Spending is cumulative and monthly*

The bolded interaction term in Formula 3 helped us determine whether the correlation between the drug utilization of the two age groups and televised advertising spending was significantly different. For each drug, “Patient Count” in Formula 3 includes both age groups. We assigned either “0” or “1” values to the “dummy” variable for the older and younger age groups, respectively. If the *p*-value of the interaction term coefficient was less than 0.01, then the two age groups expressed significantly different drug utilization linked to advertising spending; if not, the difference between age groups was insignificant (Supplementary Table 2).

In order to compare drug utilization correlations between age groups, we added a baseline cumulative spending of $100,000 to our initial regression models and generated utilization data as a function of cumulative advertising spending. To compare drug utilization between the two age groups as a function of advertising spending, we divided the count of the larger change in patient group utilization by the count of the smaller utilization change. With this, we could quantify the relative strength of each group’s drug utilization trend for each drug that displayed a significant age-related difference in utilization associated with advertising spending (Table 2).

## RESULTS

We observed that total spending for televised pharmaceutical advertisements grew by more than $1 billion over the ten-year span, a roughly 44%increase (Fig. 1). The surge was carried by the last three years of the study period—2015, 2016, and 2017.

Although advertising spending accelerated, the total number of advertised drugs changed little during the study period (Fig. 1). Thus, we can observe that television advertising spending per branded drug has increased substantially, especially in recent years.

For 13 of the 14 drugs we analyzed, the utilization behavior of the two patient subpopulations moved in concert with one another. That is, when total utilization was increasing, utilization by both seniors and the under-65 population increased; when total utilization was decreasing, utilization by both age groups decreased. Aripiprazole was the only drug with a conflicting utilization pattern: as patients under 65 were decreasing their utilization, patients older than 65 used the drug in greater numbers (Table 2).

Our first set of regression models demonstrated that, for each of the 14 drugs, and both patient age groups, there was a statistically significant correlation between utilization and cumulative monthly televised advertising spending, at a *p* < 0.01 level (Supplementary Table 1). Our second set of regression models showed that, for seven of the 14 drugs, the difference between the two patient groups’ utilization associated with cumulative monthly televised advertising spending was statistically significant at a *p* < 0.01 level (Supplementary Table 2). These seven drugs were: all three drugs in the Mood and Mental Health treatment group, three of four drugs in the Arthritis treatment group, and one of two drugs in the High Cholesterol treatment group. None of the five drugs in the Diabetes (Non-Insulin) treatment group showed a statistically significant difference in utilization by age bracket (Table 2).

For all seven drugs with significant age-related differences in utilization, the correlation between cumulative televised advertising spending and drug utilization in the 65-and-older subpopulation was significantly stronger than for patients under 65. When overall utilization of these drugs was increasing, the percentage change in seniors’ utilization was significantly greater than the percentage change in younger patients’ utilization. Similarly, when overall utilization of these drugs was decreasing, the percentage change in utilization of patients 65 and older was significantly smaller than the percentage change in utilization of patients younger than 65.

For these seven drugs, in other words, seniors demonstrated significantly greater spending-linked utilization across the study period, no matter the drug’s overall utilization trajectory. On one hand, television advertising spending was associated with seniors enrolling more vigorously when overall utilization of a drug was increasing. For instance, spending for the bipolar treatment lurasidone was associated with a growth in prescriptions 3.5 times greater among seniors compared to patients under 65 (Table 2). At the same time, our findings associated advertising spending for a drug during periods of decreasing overall utilization with seniors reducing their prescriptions more slowly than patients under 65: utilization of the arthritis drug celecoxib by patients under 65 dropped 3.3 times faster compared to older patients’ utilization (Table 2).

## DISCUSSION

For all 14 drugs we examined, we observed a sig-nificant correlation between cumulative televised ad-vertising spending and the drug utilization in both patient age groups. This finding aligns with prior research demonstrating that direct-to-consumer adv-ertising spending increases drug utilization [5, 15–18].

We found that televised advertising for certain drugs linked to significantly stronger drug utilization among seniors, compared to younger patients. For these seven drugs, the stronger age-related correlation applied both to periods of increasing and decreasing overall utilization. Although we cannot assert that advertising causes seniors to respond with greater drug utilization, the stronger correlation between spending and seniors’ utilization of certain drugs does suggests that televised advertising of prescription drugs may influence seniors more than younger viewers. Considering the cognitive decline [24, 25, 27], greater medication usage [20, 29, 30], and greater consumption of television among senior populations [19], the correlation we found highlights the potential of televised direct-to-consumer advertising to unduly influence seniors’ drug utilization behavior.

Several factors limit what our findings may assert about the relationship between televised advertising spending and the drug utilization of seniors. Our regressions do not account for channels of direct-to-consumer advertising other than television, which, although challenged increasingly by the Internet as a pharmaceutical advertising platform [1], channeled an impressive growth in advertising spending during the study period (Fig. 1). It is possible, however, that younger patients may have better responded to advertisements delivered through social media or other platforms. Nor did our study cover pharmaceutical promotion directed toward healthcare providers, such as physician detailing, free samples, and scientific journal advertisements. Including these predictors with our direct-to-consumer advertising data would return a more balanced, robust, and correctly specified model. Furthermore, we do not know what per-centage of our cohort was exposed to televised drug advertisements, nor how many discussed a prescription with their physician after viewing advertisements. Finally, we recognize the possibility that increased patient utilization and drug sales may also, in turn, encourage further advertising spending for that drug. These factors limit us from claiming a causal relationship between advertising spending and drug utilization, as previous studies have [5, 15–18].

Limitations may also arise from the patient population and drugs we elected to study. Compared to younger patients, physicians may be more reluctant to discontinue a senior’s prescription if it is working. Such therapeutic inertia could explain why seniors decreased utilization of three drugs (etanercept, celecoxib, atorvastatin) more slowly than younger patients, but it would not explain seniors’ faster uptake observed in four other drugs (lurasidone, aripiprazole, duloxetine, adalimumab) (Table 2). Moreover, relying on Medicare data means that our under-65 population—composed of patients with permanent disabilities, amyotrophic lateral sclerosis, or end-stage renal disease [32]—is not a representative sample of under-65 Americans. Compared to the overall under-65 population, Medicare patients younger than 65 may exhibit different drug utilization patterns or engage differently with drug advertisements. Future research may benefit from more granular population data, incorporating factors such as race and geography to more precisely study how direct-to-consumer advertising may affect different demographics.

Given that our study only includes drugs advertised directly to consumers, we cannot compare changes in utilization between advertised and non-advertised drugs within a therapeutic group. We also considered that the stronger correlation between advertising spending and seniors’ drug utilization could be caused by the age-related prevalence of diseases treated with the drugs in our study. That is, we considered whether seniors might display stronger correlations for drugs treating diseases more prevalent in older populations, and weaker correlations for drugs treating diseases less prevalent in older populations. If the age-related prevalence of a disease did explain the correlation between advertising spending and utilization of drugs treating that disease, then we would expect to find that patients in the more susceptible age group express significantly greater utilization for all drugs treating that disease.

As Table 2 illustrates, the data do not fully support that explanation. In fact, although depression [37] and bipolar disorder [38] are more frequently diagnosed in younger age groups, our study found a stronger correlation between advertising spending and seniors’ utilization of all three drugs in the mental health treatment group (Table 2). One should note, however, that antipsychotics such as aripiprazole are also frequently prescribed to elderly patients for conditions including dementia [39, 40]. Additionally, seniors are disproportionately affected by type 2 diabetes [41], but all five of the diabetes drugs showed comparable utilization rates linked to advertising spending between age groups (Table 2). With the high cholesterol drugs in our study, which are more likely to be prescribed to seniors than younger patients [42], only one of the two drugs’ spending was associated with significantly higher drug utilization in seniors (Table 2). Moreover, past study demonstrates that direct-to-consumer advertising does positively impact the utilization of cholesterol-lowering statins [5].

Finally, although increases in advertising spending for three of four drugs treating arthritis—an affliction more prevalent in seniors [43]—correlated to significantly stronger senior utilization, spending for the arthritis drug tofacitinib did not (Table 2). Thus, we did not find that all drugs in any treatment group uniformly exhibited stronger spending-related utilization by the age group more likely to be treated for the corresponding disease or ailment. Drugs that treat an older-skewing disease may be used at higher rates among older patients, but our findings do not suggest that the age profile of a disease explains why advertising spending for certain drugs is linked to greater utilization by older patients.

In addition to the age-related differences between patient utilization, therefore, our findings lend some nuance to the landscape of prescription drugs. The differences we observed between treatment groups may propose further study in order to understand how the consequences of direct-to-consumer advertising vary according to the advertised drug. Paired with a more granular understanding of prescription drug use, this study may encourage advertising regulations to consider for the treatment group of an advertised drug.

The stronger correlation we observed between televised advertising spending in certain drug categories and seniors’ drug utilization rates raises concerns about the influence of direct-to-consumer advertising on seniors. These findings propose further investigation into how direct-to-consumer advertising shapes the drug utilization behaviors of elderly patients. More robust models evaluating how prescription drug advertising contributes to seniors’ drug utilization—in addition to inappropriate prescriptions and adverse drug reactions—will help inform relevant regulatory and policy debates, in addition to clinical best practices.

Direct-to-consumer advertising policy recommen-dations could potentially take into account age-related cognitive deficiencies, such as in regulating the delivery of risk and side effect information, the mode of advertising, or advertising frequency. Our findings complement past research demonstrating that older individuals with cognitive deficits display less capable medical decision-making [44] and medication adherence [45].

Our findings also suggest age-conscious clinical guidelines for drug prescription policies. The influence of direct-to-consumer advertising on seniors may be mitigated by proactive reviews of older patients’ drug treatment regimens, particularly as many patients accrue drug prescriptions with age [20, 29, 30]. Likewise, patient requests for specific drug brands may prompt physicians to inquire about and discuss a patient’s information source, in addition to underlying symptoms.

Our findings may be particularly important to physicians treating Alzheimer’s disease. Most Alz-heimer’s disease patients are above age 65 [46], and the study concludes that television advertising can have an outsized impact on that age population. Physicians treating Alzheimer’s disease patients should be aware of this result, in light of the range of drugs their patients may be taking for other related and unrelated disease states. Of particular importance, some of the strongest correlations we found were for the mood and mental health disorder drugs, lurasidone, aripiprazole, and duloxetine. With direct-to-consumer television advertising, the response percentage of older patients who were on lurasidone, aripiprazole, and duloxetine increased by 3.5, 2.5, and 1.6 times the response percentage of younger patients taking these drugs. Neuropsychiatric symptoms are common in Alzheimer’s disease patients. In addition, the disease states treated by these drugs are several times higher for patients with dementia than for patients without dementia [47, 48], and neuropsychiatric symptoms, in general, are common in patients with Alzheimer’s disease [49, 50] and diseases associated with dementia. Thus, this finding should be of particular interest for physicians treating Alzheimer’s disease and dementia patients.

Clinical prescription guidelines could complement existing spending transparency rules to better situate physicians as mediators of the drug information advertised directly to consumers. Recognizing the clinical implications of this research will help ensure that advertising improves health care access and literacy without exploiting vulnerable populations, such as the elderly, in the process. Drawing on past research of age-related cognitive decline and the effects of direct-to-consumer spending, we find here that televised pharmaceutical advertising may have an outsized impact on senior citizens.

## Supplementary Material

Supplementary Material
