# Supplementary material for: Physicians Treating Alzheimer’s Disease Patients Should Be Aware that Televised Direct-to-Consumer Advertising Links More Strongly to Drug Utilization in Older Patients
Source: J Alzheimers Dis. 2021 Jun 1;81(3):1169–79. doi: 10.3233/JAD-210294 (PMC8293633; doi:10.3233/JAD-210294)
Supplement: Supplementary Material [file jad-81-jad210294-s001.pdf]

Supplementary Material

Physicians Treating Alzheimer's Disease Patients Should Be Aware that Televised Direct-to-Consumer Advertising Links More Strongly to Drug Utilization in Older Patients

Supplementary Table 1. Negative binomial regression results: significance between utilization and monthly televised direct-to-consumer advertising spending.*

Drug Name	Age Group	β_1 (SE_{β_1})†	β_2 (SE_{β_2})‡	Deviance§	*df*	GoF**	No. Obs. ††
**Lurasidone**	Older than 65	2.63e-06*** (1.78e-07)		5.77	46	0.13	48
	Younger than 65	7.60e-07*** (1.59e-07)		7.55	46	0.16	48
**Aripiprazole**	Older than 65	2.13e-06*** (6.62e-08)		15.38	89	0.17	91
	Younger than 65	1.05e-06*** (2.59e-07)	-2.0e-12*** (4.51e-13)	6.32	88	0.07	91
**Duloxetine**	Older than 65	2.48e-06*** (1.88e-07)	-1.0e-12*** (2.18e-13)	28.71	89	0.32	92
	Younger than 65	1.60e-06*** (1.87e-07)	-1.4e-12*** (2.17e-13)	33.74	89	0.38	92
**Adalimumab**	Older than 65	2.05e-06*** (2.81e-07)	-2.3e-12*** (4.17e-13)	32.49	94	0.35	97
	Younger than 65	1.26e-06*** (2.74e-07)	-1.6e-12*** (4.08e-13)	30.51	94	0.32	97
**Etanercept**	Older than 65	7.29e-07*** (2.52e-07)	-1.6e-12*** (3.78e-13)	33.65	122	0.28	125
	Younger than 65	-5.5e-07** (2.51e-07)	-1.3e-12*** (3.78e-13)	46.06	122	0.38	125
**Tofacitinib**	Older than 65	7.73e-06*** (7.62e-07)	-1.3e-11*** (1.57e-12)	39.54	52	0.76	55
	Younger than 65	8.20e-06*** (7.58e-07)	-1.4e-11*** (1.56e-12)	70.11	52	1.35	55
**Celecoxib**	Older than 65	-5.4e-07*** (8.26e-08)		14.42	90	0.16	92
	Younger than 65	-2.0e-06*** (3.34e-07)	2.25e-12*** (7.18e-13)	24.33	89	0.27	92
**Dulaglutide**	Older than 65	2.26e-05*** (1.50e-06)	-6.2e-11*** (6.29e-12)	35.15	24	1.46	27
	Younger than 65	2.47e-05*** (1.57e-06)	-6.7e-11*** (6.53e-12)	45.14	24	1.88	27
**Liraglutide**	Older than 65	3.07e-06*** (8.67e-07)	-8.8e-12*** (3.28e-12)	8.45	49	0.17	52
	Younger than 65	1.03e-06*** (2.05e-07)		19.88	50	0.4	52
**Dapagliflozin**	Older than 65	3.86e-06*** (1.26e-06)	1.23e-11*** (4.06e-12)	11.49	37	0.31	40
	Younger than 65	8.27e-06*** (3.01e-07)		21.08	38	0.55	40
**Empagliflozin**	Older than 65	3.22e-05*** (2.54e-06)	-1.0e-10*** (1.47e-11)	29.25	25	1.17	28
	Younger than 65	1.55e-05*** (6.50e-07)		72.48	26	2.79	28

**Canagliflozin**	Older than 65	1.64e-05*** (1.16e-06)	-6.0e-11*** (5.32e-12)	25.21	39	0.65	42
	Younger than 65	1.95e-05*** (1.17e-06)	-7.1e-11*** (5.36e-12)	29.02	39	0.74	42
**Atorvastatin**	Older than 65	-1.8e-06*** (2.62e-07)	1.53e-12*** (4.18e-13)	8.42	64	0.13	67
	Younger than 65	-2.0e-06*** (2.63e-07)	1.42e-12*** (4.19e-13)	12.4	64	0.19	67
**Rosuvastatin**	Older than 65	2.44e-06*** (1.35e-07)		11.17	65	0.17	67
	Younger than 65	2.18e-06***(1.36e-07)		14.6	65	0.22	67

*Supplementary Table 1 exhibits that there is a significant correlation, for both patient age groups, between drug utilization and direct-to-consumer advertising spending. The table, in specific, shows the results of the first set of negative binomial regressions along with the coefficients' significance, standard error, deviance, degree of freedom, goodness of fit, and the number of observations. This analysis also helped us compare how the percentage change in drug utilization varied between each patient age group.

†Where: ***p <= 0.01, **p <= 0.05, *p <= 0.1, p > 0.1

‡For empty cells, the addition of the quadratic term was found not to significantly improve upon the model and thus, not included.

§The error or the difference between the actual and the predicted data. The larger it is, the worse the model estimations become. In addition, it is used as a factor to test goodness of fit among regression models.

**The result of dividing the model deviance by its Degrees of Freedom *(df)*. The closer it is to 1, the better the model estimation becomes.

††Equivalent to the number of months between the first instance of televised ad spending and the last instance of spending or the first instance of generic competition. A generic competitor is defined as a drug with the same active ingredient, dosage strength, and form as the brand drug, produced by a different manufacturer.

Supplementary Table 2. Negative binomial regression results: significant difference in utilization between patients 65 and older and patients younger than 65.*

Drug Name	β_1 (SE_{β_1})†	β_2 (SE_{β_2})	β_3 (SE_{β_3})	β_4 (SE_{β_4})	Deviance	*df*	GoF	No. Obs.
**Lurasidone**	2.15393*** (0.05528)	3.47e-06*** (5.85e-07)	-1.8e-06*** (2.39e-07)	-2.2e-12. (1.51e-12)	11.09	91	0.12	96
**Aripiprazole**	2.34824*** (0.0328)	2.95e-06*** (1.92e-07)	-2.2e-06*** (9.25e-08)	-1.4e-12*** (3.22e-13)	21.68	177	0.12	182
**Duloxetine**	0.90801*** (0.03084)	2.67e-06*** (1.37e-07)	-1.2e-06*** (6.50e-08)	-1.2e-12*** (1.54e-13)	64.52	179	0.36	184
**Adalimumab**	0.37142*** (0.02457)	1.81e-06*** (2.01e-07)	-3.2e-07*** (8.87e-08)	-2.0e-12*** (2.91e-13)	64.45	189	0.34	194
**Etanercept**	0.33538*** (0.02666)	6.17e-07*** (1.82e-07)	-1.0e-06*** (7.47e-08)	-1.4e-12*** (2.67e-13)	80.12	245	0.33	250
**Tofacitinib**	0.01006. (0.06292)	7.93e-06*** (5.52e-07)	7.76e-08. (2.57e-07)	-1.3e-11*** (1.11e-12)	109.8	105	1.05	110
**Celecoxib**	-1.17175*** (0.02742)	-1.2e-06*** (2.42e-07)	-4.4e-07*** (1.17e-07)	1.47e-12*** (5.04e-13)	40.21	179	0.22	184
**Dulaglutide**	-0.35496*** (0.05882)	2.32e-05*** (1.10e-06)	8.93e-07* (4.64e-07)	-6.4e-11*** (4.53e-12)	80.61	49	1.65	54
**Liraglutide**	-0.1626*** (0.0391)	2.76e-06*** (6.31e-07)	2.47e-07. (2.89e-07)	-7.6e-12*** (2.32e-12)	24.81	99	0.25	104
**Dapagliflozin**	-0.21976*** (0.07821)	4.54e-06*** (9.34e-07)	6.33e-07. (4.13e-07)	1.01e-11*** (2.93e-12)	29.95	75	0.4	80
**Empagliflozin**	-0.49246*** (0.07435)	3.09e-05*** (1.92e-06)	7.50e-07. (9.21e-07)	-9.7e-11*** (1.08e-11)	71.4	51	1.4	56
**Canagliflozin**	-0.19826*** (0.04752)	1.75e-05*** (8.48e-07)	7.61e-07* (3.93e-07)	-6.6e-11*** (3.77e-12)	56.23	79	0.71	84
**Atorvastatin**	-1.03427*** (0.03061)	-1.7e-06*** (1.91e-07)	-2.9e-07*** (9.37e-08)	1.48e-12*** (2.96e-13)	20.86	129	0.16	134
**Rosuvastatin**	-1.05377*** (0.03725)	3.29e-06*** (4.71e-07)	-2.5e-07. (1.91e-07)	-2.4e-12* (1.27e-12)	22.23	129	0.17	134

*Supplementary Table 2 shows the result of the second set of negative binomial regressions, including the bolded interaction term used in Formula 3. The crucial value is the significance level of the third coefficient—β_3—represented in the table's bolded fourth column. Three asterisks beside the coefficient indicates a significant, age-based difference in utilization associated with advertising spending for that drug. Such significance can be observed for 7 of the 14 drugs.

†Where: ***p <= 0.01, **p <= 0.05, *p <= 0.1, p > 0.1
